# Balancing the interplay of histone deacetylases and non-coding genomes: a step closer to understand the landscape of cancer treatment

**DOI:** 10.1186/s12920-023-01724-3

**Published:** 2023-11-17

**Authors:** Jingjing Pu, Ting Liu, Amit Sharma, Ingo G. H. Schmidt-Wolf

**Affiliations:** 1 Department of Integrated Oncology, Center for Integrated Oncology (CIO) Bonn, University Hospital Bonn, Bonn, Germany; 2 Translational Biogerontology Lab, German Center for Neurodegenerative Diseases (DZNE), Venusberg-Campus 1/99, 53127 Bonn, Germany

**Keywords:** Histone deacetylases, Non-coding genome, Epigenetics, Cancer

## Abstract

Histone deacetylase (HDAC) inhibitors have enormous therapeutic potential as effective epigenetic regulators, and now with the focus on the selective HDAC6 inhibitor in ongoing clinical trials, more advantages over other non-selective pan-HDAC inhibitors are foreseeable. As it is of paramount importance to understand the complex regulatory web of mutual interactions involving epigenetic machinery and non-coding genome in regulating gene expression, herein, we investigated the intriguing interactions between HDAC6-induced lncRNA (LINC00152) and its possible sponge miRNA (hsa-miR-499a-5p) in multiple myeloma.

**Supplementary Information:**

The online version contains supplementary material available at 10.1186/s12920-023-01724-3.

## Main text

The intriguing interactions between the non-coding genome and the epigenetic machinery that regulate gene expression have preoccupied researchers over the past decades. Certainly, the question of how organisms/cells have adapted to these multiple regulatory mechanisms continues to resurface. Despite the fact that the functional existence of microRNAs relies exclusively on miRNA-mRNA interactions, another obvious concern is why the crosstalk of lncRNAs and microRNAs is so pivotal and what is the rationale behind the involvement of epigenetic enzymes in this complexity. With recent advances, it is undeniable that a deregulated noncoding genome poses a critical factor in diseases [1], particularly cancer, and epigenome mapping has highlighted that certain patient cell populations can be sensitive to drugs and therapies [2]. Thus, the mutual interactions between the noncoding genome and the epigenetic machinery exert their biological functions in the dysregulated genome.

**Fig. 1:**
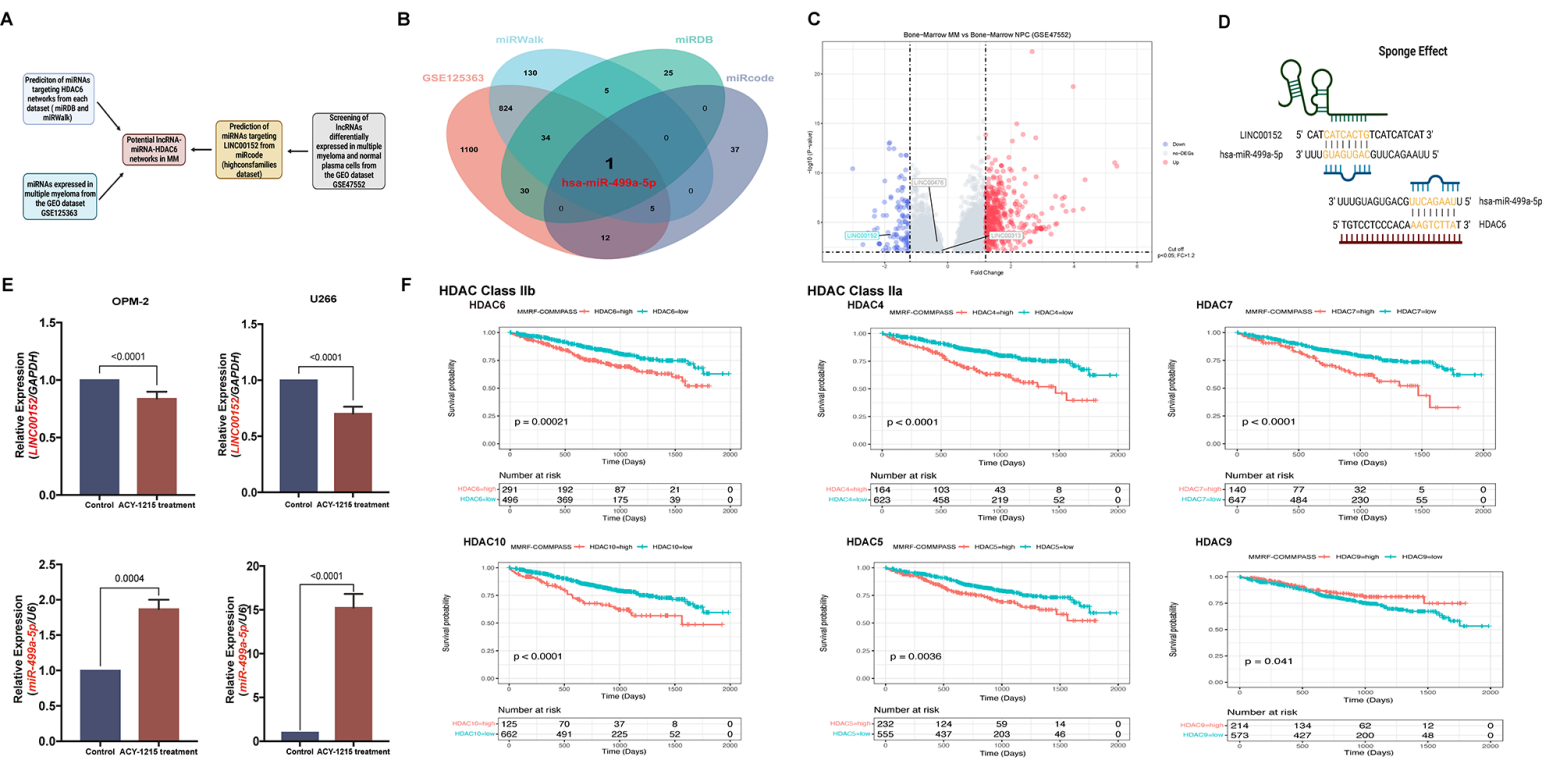
HDAC6- LINC00152- hsa-miR-499a-5p network in multiple myeloma. (**A**) Workflow for the identification of potential regulatory LINC00152 miRNA-HDAC6 networks. (**B**) Overlapping miRNA was identified using relevant public databases (GSE125363, miRDB, miRWalk and prediction of miRNAs targeting LINC00152 from miRcode (highconsfamilies dataset)). (**C**) Volcano plot for screening the lncRNAs differentially expressed in multiple myelomas obtained from GEO dataset GSE47552. (**D**) LINC00152 acted as a sponge for hsa-miR-499a-5p in MM cells. Schematic representation of the binding sites between has-miR-499a-5p and HDAC6 3’UTR. (**E**) Relative mRNA expression levels of LINC00461 and hsa-miR-499a-5p in MM cell lines (OPM-2 and U266) treated with either DMSO or 2 µM HDAC6 inhibitor (ACY-1215). Results represent data from three separate experiments. Data are presented as mean ± standard deviation (SD). (p < 0.05, unpaired Student’s t-test). (**F**) HDAC class IIb (HDAC6 and HDAC10) and HDAC class IIa (HDAC4, HDAC5, HDAC7 and HDAC9) showed significant prognostic ability in multiple myeloma retrieved from MMRF-COMMpass database

Since it is of utmost importance to understand the complex regulatory web of these mutual interactions, Wu and colleagues presented some interesting results on this concept using glioblastoma model [3]. The authors successfully confirmed the altered expression of lncRNA (LINC00461) after inhibition of histone deacetylase 6 (HDAC6) and also identified the interaction of HDAC6 and RNA-binding proteins in regulating its stability. A further section to be appreciated was the methodology for predicting lncRNA-miRNA mRNA networks, which prompted us to try a similar approach in multiple myeloma (MM). We directly used reliable publicly available MM datasets and identified an HDAC6-induced lncRNA (LINC00152) and its possible sponge miRNA (hsa-miR-499a-5p) (Fig. 1, supplementary file 1). Interestingly, we confirmed that the clinically applicable HDAC6 inhibitor (ACY-1215/Ricolinostat) was capable of inducing alterations in the expression of LINC00152 and hsa-miR-499a-5p in MM cell lines (OPM-2 and U266). To determine whether targeting HDAC6 and its non-coding network is vulnerable in the clinic, we further examined the expression pattern of the HDAC family in MM and found both members of HDAC class IIb (HDACs 6 and 10) as prognostically relevant. Except for some (HDAC11, SIRT2 and SIRT4), several other members of the HDAC family also showed prognostic significance in MM (Supplementary Fig. 1).

Undeniably, HDAC6 as a selective inhibitor has more advantages over other non-selective pan-HDAC inhibitors [4]. While the availability of newer HDAC6 selective inhibitors for the treatment of MM is exciting [5, 6], so is the synergistic compatibility of HDAC6 with non-oncology drugs (e.g., Meticrane) [7], raising the possibility of broader clinical application. Being a pioneer in cytokine-induced killer cell (CIK) immunotherapy, we have already demonstrated the beneficial effect of CIK cells with HDAC inhibitors against MM cells, and therefore HDAC6-specific clinical trials in this context can reasonably be anticipated in the future [8]. Independently, the role of the non-coding genome in MM, which has long been underappreciated, is increasingly being recognized [9, 10, 11]. Therefore, it will be of future interest to find out whether the integrated network of HDAC6, non-coding genome, and targeting mRNAs has any potential overlaps in cancer types (e.g. in MM) or diseases in general [12]. Nevertheless, the involvement of HDACs (especially HDAC6) in inhibiting or promoting cancer development and progression is becoming more apparent [12. Ongoing research to identify the underlying mechanisms is, of course, also receiving a boost [13]. Similar scenario is also quite apparent for non-coding genome [14, 15]. Now the significant work by Wu and colleagues has added an additional layer of information by offering insights into the HDACs- lncRNA-miRNA-mRNA axis. It is now foreseeable that more studies using the same axis in different cancer types may help to find a common module with anticancer potential.

### Electronic supplementary material

Below is the link to the electronic supplementary material.

Supplementary Material 1

Supplementary Material 2

Supplementary Material 3

## Data Availability

Not applicable.

## Abbreviations

HDAC: Histone deacetylates
lncRNAs: Long noncoding RNAs
miRNAs: MicroRNAs
