# Supplementary figures and images for: Balancing the interplay of histone deacetylases and non-coding genomes: a step closer to understand the landscape of cancer treatment
Source: BMC Med Genomics. 2023 Nov 17;16:295. doi: 10.1186/s12920-023-01724-3 (PMC10657130; doi:10.1186/s12920-023-01724-3)

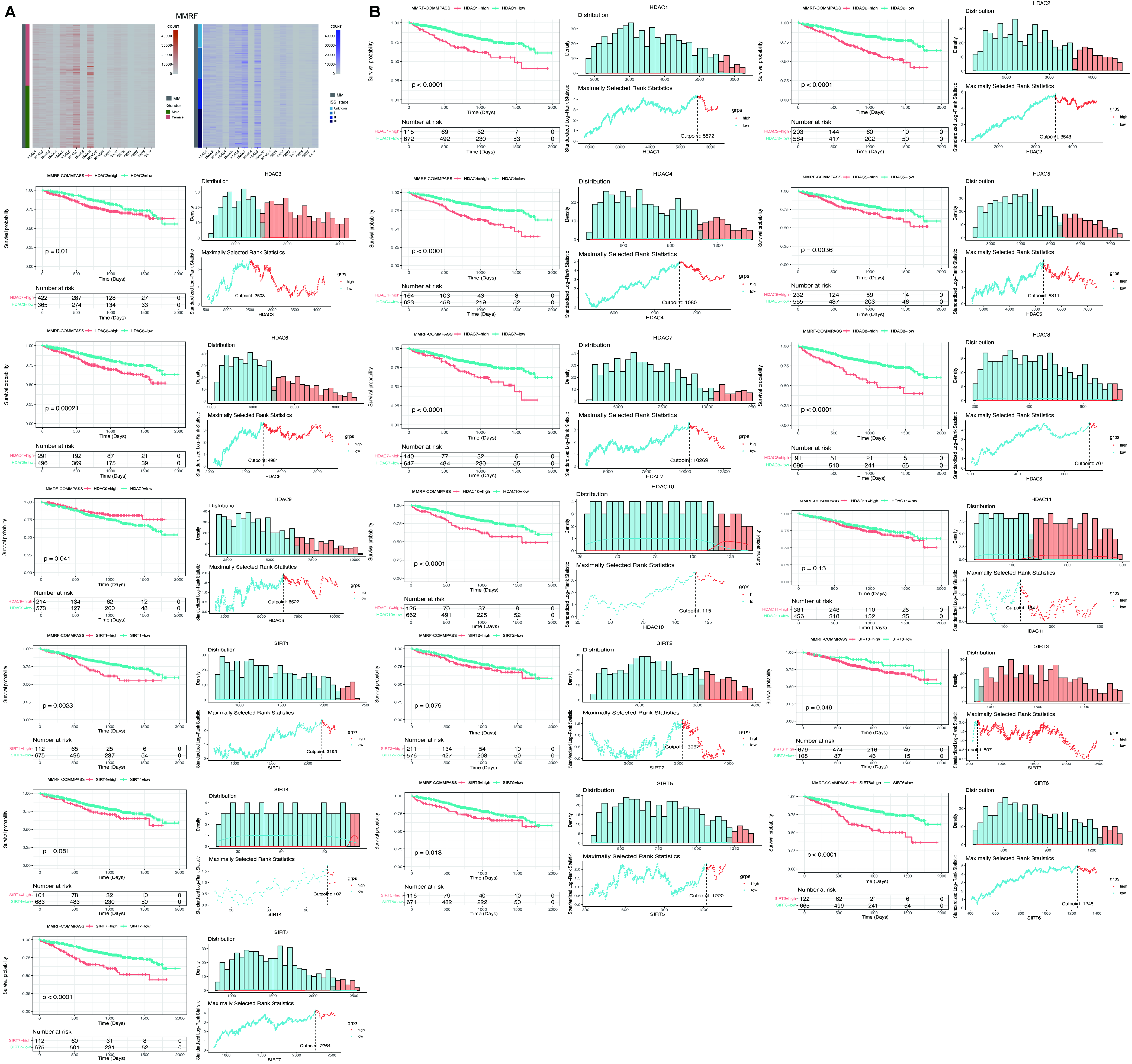

Supplement: Supplementary file 1 — Supplementary Material 1 [file 12920_2023_1724_MOESM1_ESM.tif]
