# Supplementary material for: Balancing the interplay of histone deacetylases and non-coding genomes: a step closer to understand the landscape of cancer treatment
Source: BMC Med Genomics. 2023 Nov 17;16:295. doi: 10.1186/s12920-023-01724-3 (PMC10657130; doi:10.1186/s12920-023-01724-3)
Supplement: Supplementary file 2 — Supplementary Material 2 [file 12920_2023_1724_MOESM2_ESM.docx]

**Supplementary figure 1 legend**

A. Heatmaps of the expression pattern of the HDAC family in the MMRF-COMMpass dataset across genders and ISS stages. B. Kaplan–Meier survival analysis of the HDAC family in MMRF-COMMpass dataset, along with cutpoint selection. The x-axis represents overall survival time, and the y-axis represents the overall survival rate. P < 0.05 is considered statistically significant. The optimal cutpoint was determined using the maximally selected rank statistics.
