# Supplementary material for: Balancing the interplay of histone deacetylases and non-coding genomes: a step closer to understand the landscape of cancer treatment
Source: BMC Med Genomics. 2023 Nov 17;16:295. doi: 10.1186/s12920-023-01724-3 (PMC10657130; doi:10.1186/s12920-023-01724-3)
Supplement: Supplementary file 3 — Supplementary Material 3 [file 12920_2023_1724_MOESM3_ESM.docx]

**Supplementary file 1**

**Methods and Materials**

*RNA‐Seq data collection*

RNA-seq data from the GSE125363 and GSE47552 datasets were retrieved from the comprehensive gene expression (GEO) database to filter out miRNAs and lncRNAs relevant to multiple myeloma onset and development. The miRNA expression profile of the GSE125363 dataset was retrieved using the GPL18044 platform (Agilent-046064 Unrestricted_Human_miRNA_V19.0_Microarray (Feature Number Version)). The lncRNA expression profile of the GSE47552 dataset was retrieved using the GPL6244 platform ([HuGene-1_0-st] Affymetrix Human Gene 1.0 ST Array [Transcript (Gene) Version]). The Cancer Genome Atlas (TCGA) (https://portal.gdc.cancer.gov) provided the data for MMRF-COMMpass. In the study, there were 48 and 99 samples selected for investigation from the GSE125363 and GSE47552 datasets, respectively, including patients with multiple myeloma and healthy individuals. In addition, 709 MMRF-COMMpass cases were selected for further investigation.

*Data Processing and DEG Identification*

R version 4.3.1 was used for all analyses in this study. DEGs were detected between MM patients and healthy donors using the R package "limma". Genes with P < 0.05 and [log2FoldChange (log2FC)] > 1.2 were considered DEGs.

*Construction of* *lncRNA/miRNA/HDAC6 axis*

GEO and TCGA datasets were used to build a network between LncRNA-miRNA-HDAC6, and interactive networks were created using the R package "igraph".

*Venn Diagram of HDAC6 related miRNA and the sponge effect of lncRNA/miRNA/HDAC6*

The intersections of central HDAC6-associated miRNAs in multiple myeloma derived from GSE125363, the miRWalk database (http://mirwalk.umm.uni-heidelberg.de/), the miRDB database (http://mirdb.org/) and the miRcode database (http://mircode.org/) are considered to be a miRNA hub more strongly associated with multiple myeloma (MM) occurrence and progression. The R package "venndiagram" was used to create a Venn diagram to obtain these intersections. LncBase Predicted v.3 (<https://diana.e-ce.uth.gr/lncbasev3>), the StarBase database (<http://starbase.sysu.edu.cn/index.php>) and TargetScanHuman V7.2 (<https://www.targetscan.org/vert_72/>) were used to predict the binding sites of lncRNA-miRNA-mRNA networks.

*Survival Analysis of HDAC family*

The R package "survival" was used to investigate the impact of different expressions of the HDAC family members on the survival of patients using RNA sequencing data and matched clinical characterstics from the TCGA.

*RT-qPCR evaluation*

RNA isolation of multiple myeloma cell line (OPM-2 and U266) was performed with RNeasy Plus Mini Kit (QIAGEN, Cat. No: 74136). Complementary DNA (cDNA) was synthesized by reverse transcription using hifiscript Kit (Invitrogen). Quantitative polymerase chain reaction was performed on LINC00152 and hsa-miR-499a-5p using P PowerTrack™ SYBR Green Mastermix (Thermo Fisher Scientific, Cat. No: A46109). The glyceraldehyde 3-phosphate dehydrogenase (GAPDH) was selected as the internal reference gene for LINC00152. The primer sequences used are as follows: LINC00152 forward 5’- AGTTACGGAGGACCCAGCAA-3’; LINC00152 reverse 5’-GGGCTGAGTCGTGATTTTCG-3’; GAPDH forward 5’-CCAGGTGGTCTCCTCTGA-3’; GAPDH reverse 5’-GCTGTAGCCAAATCGTTGT-3’. U6 was selected as the internal reference gene for has-miR-499a-5p. The primer sequence is as follows: hsa-miR-499a-5p forward 5’-GCCGAGTTAAGACTTGCAGTGA-3’; hsa-miR-499a-5p reverse 5’-CTCAACTGGTGTCGTGGA-3’; U6 forward 5’-CTCGCTTCGGCAGCACA-3’; U6 reverse 5’-AACGCTTCAGGAATTTGCG-3’. The delta–delta Ct (2^–∆∆Ct^) approach was used to measure the relative expression levels of target genes, which were standardized against GAPDH mRNA and U6 snRNA levels, respectively, for lncRNA or mRNA and miRNA.

*Statistical Analysis*

Statistical analysis was performed using GraphPad Prism (version 8.0). Experimental data are presented as means ± SD. Unpaired Student’s t-test was performed to analyze statistical significance. P < 0.05 was considered statistically significant.
